# Covalently functionalized uniform amino-silica nanoparticles. Synthesis and validation of amine group accessibility and stability†

**DOI:** 10.1039/c9na00772e

**Published:** 2020-01-16

**Authors:** Peter J. Miller, Daniel F. Shantz

**Affiliations:** Department of Chemical and Biomolecular Engineering, Tulane University 6823 St. Charles Avenue New Orleans LA 70118 USA dshantz@tulane.edu

## Abstract

This paper describes the synthesis and characterization of colloidally stable, 18 nm silica nanoparticles that are functionalized with amine groups. Electron microscopy, small-angle X-ray scattering (SAXS), and dynamic light scattering show the amine grafting does not impact particle size. SAXS and DLS confirm the particles do not aggregate at 10 mg mL^−1^ and pH 2 for 30 days. Ninhydrin analysis, fluorescamine binding, and NMR studies of carboxylic acid binding show that the amines are present on the surface and accessible with maximum loading calculated to be 0.14 mmol g^−1^. These materials should find a range of use in nanotechnology applications.

## Abstract 2

This paper describes the synthesis and characterization of stable, 18 nm silica nanoparticles that are functionalized with amine groups.

## Graphical abstract

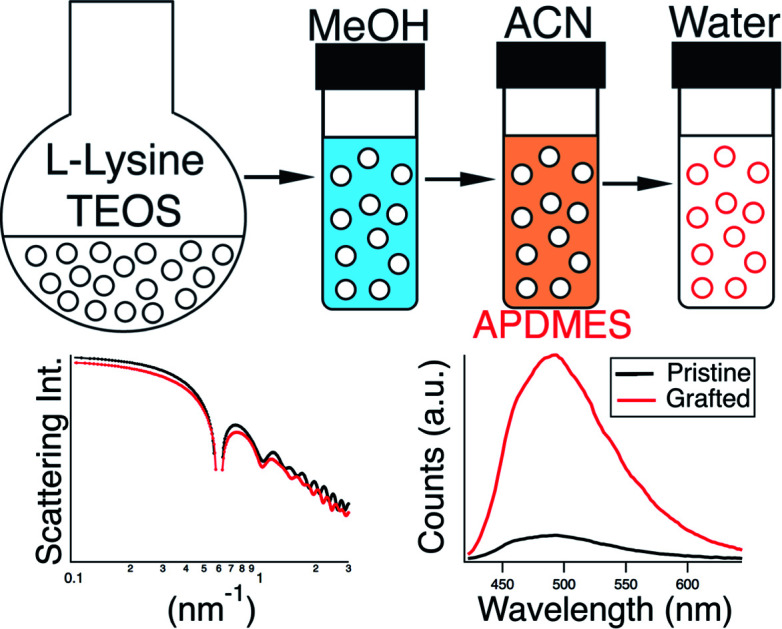

## Introduction

Silica nanoparticles have been studied for numerous applications including catalysis,^1,2^ pharmaceuticals,^3,4^ and thin films.^5,6^ Silica nanoparticles with surface modification are of great interest as potential supports for proteins,^7^ therapeutic drugs,^8–11^ fluorophores,^12,13^ active groups for separation/catalysis,^14–19^ and polymers.^13,20–22^ Surface modification also allows redispersion and colloidal stability in a variety of media,^23–25^ which has been difficult specifically for silica due to the high density of surface silanol groups causing irreversible aggregation.^26^ It has been reported that irreversible aggregation becomes more severe as particle sizes decrease,^24,27^ but in the case of mesoporous particles complete redispersion can be obtained after drying with the aid of sonication, with the smallest hydrodynamic diameter obtained approximately 40 nm.^11^ Redispersion and colloidal stability of sub-50 nm diameter non-porous silica nanoparticles have only been shown in a few instances,^28,29^ utilizing long chain functional groups that could not be quantified.

Silica nanoparticles are traditionally synthesized using the Stöber method^30^ with ammonium hydroxide as a base for silica hydrolysis and condensation. To obtain sub-20 nm particles zwitterionic amino acids are frequently used to cap the surface and control growth, as first shown by Yokoi *et al.*^31^ The resulting silica nanoparticles are capped with amino acids, but because the groups are not covalently attached, the groups are easily exchangeable and irreversible aggregation occurs at high and low pH. To avoid these drawbacks, organic functional groups have been covalently linked to the surface silanol groups using alkoxysilanes.^11,23,28,29,32^ This has shown improvement for functional group robustness and colloidal stability, but the surface functionalization must be completed in organic solvents as the alkoxy groups react with water.

Surface functionalization can be completed during synthesis by co-condensation^25,33,34^ or post-synthetically^35^ by transferring to an organic solvent, usually by first drying, then redispersing. This drying steps leads to difficulties during redispersion, especially for sub-50 nm diameter particles,^26^ and post-synthetic grafting has only been shown for that small of diameters in a few instances when using mesoporous nanoparticles functionalized with long chain polymeric functional groups.^11,24^ A key factor for colloidal stability after functionalization is colloidal stability during functionalization,^26^ which due to aggregation after synthesis, has only been possible using the co-condensation method.^28,29^ However, co-condensation has been shown to result in larger and less uniform particles.^18,34,36,37^

Herein a phase transfer method is developed to transfer l-lysine capped silica nanoparticles from water to acetonitrile while maintaining colloidal stability. The phase transfer method took advantage of the native l-lysine capping during synthesis to stabilize the particles at low pH in methanol, as first shown on HfO_2_ and ZrO_2_ when amino acid was added during redispersion.^38^ Grafting of amine groups using (3-aminopropyl)dimethylethoxysilane (APDMES) could then be performed on the colloidal particles when they were suspended in acetonitrile. Amine groups were chosen because of their well-documented catalytic,^15,19,24,39,40^ separations,^17,41^ and biological applications^42,43^ as well as the ability to react specific molecules to the amine allowing the synthesis of more complex functional groups.^44–47^ The resulting amine grafted particles could be resuspended in pH < 5 aqueous solutions with minimal diameter change from the ungrafted when characterized using transmission electron microscopy (TEM), small angle X-ray scattering (SAXS), and dynamic light scattering (DLS). Amine surface functionalization was confirmed using fluorescamine and ninhydrin assays. This work demonstrates it is possible to make stable amine-functionalized small silica nanoparticles (<20 nm) with readily accessible functional groups.

## Experimental

### Materials

All compounds were used as received. 98% l-lysine, 99% hexanoic acid, 97% 3-aminopropyltrimethoxysilane (APTMS), 95% *O*-(2-carboxyethyl)-*O*′-methyl-undecaethylene glycol (CMUG), 99% potassium phosphate monobasic (KH_2_PO_4_), and 98% potassium phosphate dibasic (K_2_HPO_4_) were purchased from Sigma-Aldrich. HPLC grade acetonitrile and 99.9% tetrahydrofuran (THF) were purchased from EMD Millipore. 99.8% methanol (MeOH), ACS grade toluene, and biotechnology grade trifluoroacetic acid (TFA) were purchased from VWR. 95% 3-aminopropyldimethylethoxysilane (APDMES) was purchased from Gelest. 99% ninhydrin, 95% fluorescamine, and 99.9% tetraethyl orthosilicate (TEOS) were purchased from Alfa Aesar. 99.8% deuterium oxide (D_2_O) was purchased from Acros Organics. 98% propylamine was purchased from TCI.

### Synthesis of silica nanoparticles

Silica nanospheres were synthesized using TEOS as the silica source and l-lysine as the base catalyst as first reported by Yokoi *et al.*^31^ and as previously shown in our lab.^48^ 0.146 g of l-lysine was dissolved in 124 mL of DI water and heated to 70 °C. 10.2 g of TEOS was then quickly added and stirred at 500 rpm for 24 hours. The solution was then allowed to cool for 15 minutes and immediately used.

### Phase transfer and amine functionalization

2 mL of the aqueous nanosphere solution was mixed with 40 mL of THF then centrifuged at 5000 rpm for 10 minutes to remove the particles from solution. The liquid was decanted, the particles mixed with 5 mL of methanol and 100 μL of TFA and then sonicated in a VWR Ultrasonic Cleaner for 1 hour. This led to a stable suspension. The suspension was then mixed with 40 mL of toluene and centrifuged at 500 rpm for 10 minutes. The liquid was decanted and the particles were mixed with 8 mL acetonitrile and sonicated for 1 hour over ice. The suspended particles were then immediately used for amine functionalization.

**Scheme 1:**
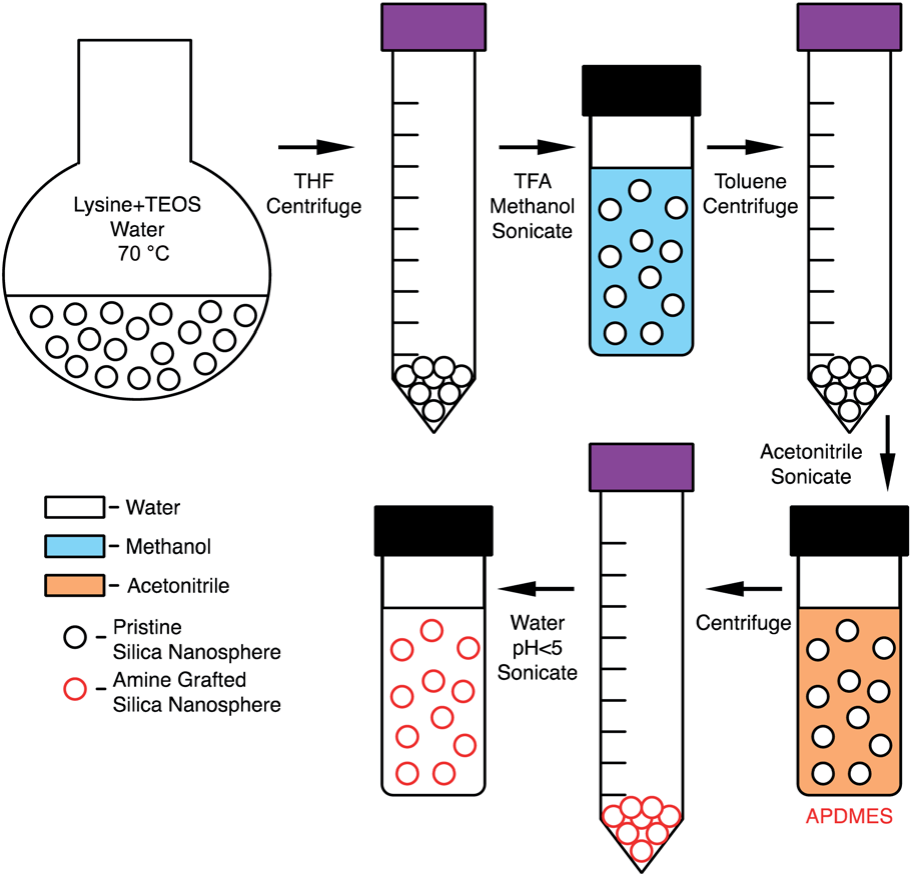
Silica phase transfer and amine grafting.

Amine functionalization was completed by adding 100 μL of 3-aminopropyldimethylethoxysilane (APDMES) to a 10 mL volumetric flask and diluting to the mark with acetonitrile. 500 μL of this solution was then added to the acetonitrile particle suspension and stirred for 1 hour. The particles flocculated from solution when amine grafting was successful. The solution was then centrifuged at 5000 rpm for 10 minutes then washed with 10 mL of acetonitrile and centrifuged again. Typically, 2.5 mL of DI water was then added, this water amount was adjusted depending on the desired particle concentration. Then 50 μL of 1 M HCl was added, and the solution was then sonicated for 1 hour to obtain a stable suspension in water. To further remove residual methanol and acetonitrile, dialysis was performed in 1 L of water for 12 hours with the water changed once after 6 hours. After dialysis 50 μL of 1 M HCl was added and briefly sonicated to ensure particle stability. The resulting solution was 10 mg mL^−1^ amine grafted nanoparticle (g-NPs) at pH 2. The phase transfer and grafting process is shown in Scheme 1.

### Amine confirmation and quantification

Nanoparticle surface functionalization was confirmed using ninhydrin and fluorescamine. For the analysis of ungrafted particles, 5 mL of the initial l-lysine/silica nanoparticle suspension was dialyzed overnight in 1 L of DI water to remove excess l-lysine. Then 100 μL of hexanoic acid was added and stirred for 8 hours to act as a stabilizer for the nanoparticles. The excess hexanoic acid was then removed by dialysis in 1 L of DI water and stirred overnight. In the case of g-NPs, the APDMES loading was varied by changing the volume of APDMES during the grafting step in acetonitrile. The final particles suspended in water were dialyzed before amine confirmation to remove HCl, then 100 μL of hexanoic acid was added, stirred for 8 hours, and removed by dialysis in water overnight.

For ninhydrin analysis, the grafted and ungrafted suspended nanoparticles were first dried by slow evaporation for 24 hours at 80 °C. Then, 25 mg of dry nanoparticles were added to 2 mL methanol and heated to 65 °C in a capped 8 mL vial. 1 mL of 0.2 M (356 mg in 10 mL) ninhydrin in methanol was then added and stirred at 65 °C for 1 hour. The solution was then centrifuged to remove the particles and 1 mL of the liquid was mixed with 1 mL of methanol for UV-vis analysis. The amount of accessible amine groups was calculated from a calibration curve made by using 50, 100, 250, and 500 μL of 0.01 M propylamine stock in methanol. The initial 2 mL of methanol was reduced by the difference of how much propylamine stock was added, *i.e.* for 500 μL of propylamine stock only 1.5 mL of methanol was used during the ninhydrin reaction step. The stock solutions were used as is after the reaction and 1 mL was mixed with 1 mL of methanol for UV-vis analysis. For all samples, the visible spectrum was scanned from 500 to 700 nm with the peak height at 580 nm used for quantification.

For fluorescamine analysis the suspended particles were immediately used after dialysis and 100 μL of solution was added to a microplate well. Then, 100 μL of 0.1 M potassium phosphate buffer (1.64 g K_2_HPO_4_/0.081 g KH_2_PO_4_ in 100 mL DI water) and 100 μL of 1.8 mM fluorescamine (5 mg in 10 μL) in acetonitrile were added to the same well as the particle suspension. The microplate was then analyzed in a Biotek Synergy H1 fluorescence microplate reader with a 392 nm excitation wavelength. The emission wavelength was detected from 350 to 700 nm.

### NMR measurements

Particles were initially prepared at 20 mg mL^−1^ then dialyzed twice in 1 L DI water for 12 hours each. Then the solution was adjusted to pH 3 with 0.1 M HCl and sonicated for 1 hour to ensure complete dispersion. The resulting suspension was 17 mg mL^−1^ g-NPs. 100 μL of this g-NP solution was then added to the NMR tube along with 667 μL of D_2_O. 6.8 mM *O*-(2-carboxyethyl)-*O*′-methyl-undecaethylene glycol (CMUG) stock, prepared in D_2_O, was then added incrementally in 50 μL portions and when higher concentration was desired a 27.2 mM stock was used. For experiments using MeOH, a 6.8 mM stock solution was prepared in D_2_O.

### Analytical

#### Dynamic light scattering

Experiments were performed with a NanoBrook 90Plus PALS with a Brookhaven TurboCorr correlator. The wavelength of the incident beam was 660 nm and the detector angle was 90°. All samples were filtered with a 0.4 μm PTFE syringe filter (VWR) before loading into a plastic cuvette (VWR) to eliminate any dust. An elapsed time of 30 minutes was used for each sample to ensure good signal-to-noise. The measurement temperature was 25 °C and delay-time increase from 5 μs to 1 s. The intensity auto-correlation function was analysed with the non-negative constrained least squared method (NNLS).^49^ The NNLS fit yields a particle size (*d*)/translational diffusion (*D*_t_) distribution for polydisperse solutions from eqn (1) with the largest population set to 100%. The refractive index (*n*) of the silica nanoparticles was1
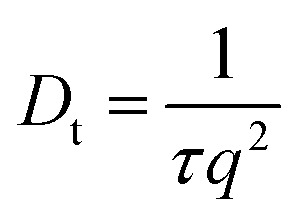
taken to be 1.43 and water 1.33. As the nanoparticle solutions were approximately 10 mg mL^−1^, particle interactions can be assumed to be negligible. The hydrodynamic diameter is calculated from *D*_t_ in eqn (1) using the Stokes–Einstein for spherical particles in water (viscosity of 0.89 cP) at 25 °C.

#### UV-vis absorption spectroscopy

UV-vis spectra were obtained from a Thermo-Fisher Evolution 300 and scanned from 500 to 700 nm at 1 nm s^−1^. The maximum absorption was observed at 580 nm and that absorption was used for all calculations.

#### Transmission electron microscopy

TEM was performed on a JEOL 2010 FEG TEM STEM at 300 kV. Nanoparticle suspensions were diluted to 0.5 mg mL^−1^ in DI water then deposited on a carbon coated Cu grid and allowed to dry for at least 1 hour. Diameter distributions were taken from 3 separate micrographs with 20 diameters recorded per image.

#### Small-angle X-ray scattering

Measurements were performed using an Anton Paar SAXSpace with Cu Kα radiation (1.5417 Å). Liquid samples were loaded into a 1 mm diameter Anton Paar quartz capillary and measured at 25 °C. The sample to detector distance was 317 mm and the measurement was performed in line mode. The scattered intensity of the nanoparticles was calculated using neat DI water as a reference solution for background subtraction. Raw scattering data was processed using Anton Paar SAXSdrive and SAXSanalysis.

#### Nuclear magnetic resonance

Measurements were performed on a Bruker Avance 500 operating at a ^1^H frequency of 500 MHz and using a BBI probe. For all measurements the sample temperature was 25 °C. All measurements used 8 scans of 32k data points and the spectral width was set to 8 ppm. T2 measurements were conducted using the Carr–Purcell–Meiboom–Gill pulse sequence, [90*x*° − *τ* − (180*y*° − 2*τ*)*n* − Acq], which uses a 180° pulse train to attenuate signals from the relaxing species.

## Results and discussion

Consistent with prior literature,^31^ using the l-lysine mediated hydrolysis of TEOS it is possible to make small, uniform silica nanoparticles. Before discussing the characterization details of the nanoparticles, a discussion of the challenges of nanoparticle functionalization are described.

**Fig. 1:**
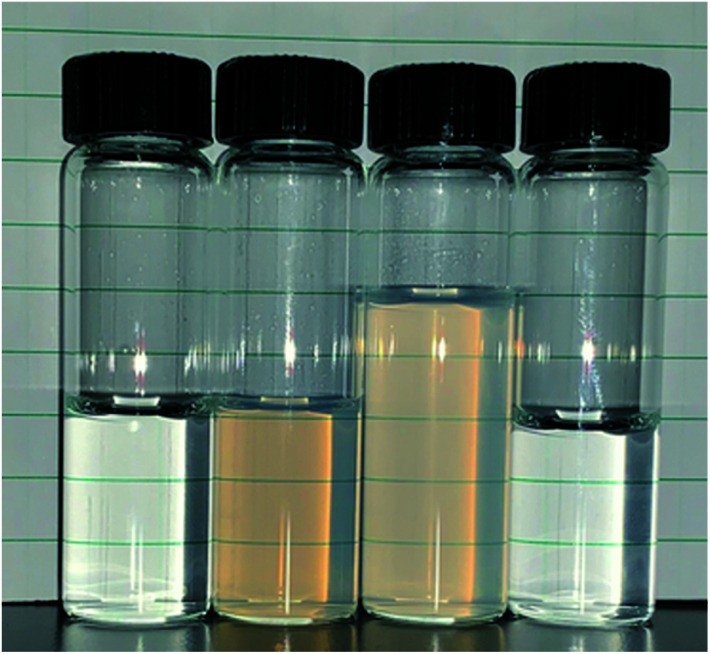
Image of silica nanoparticles suspended in each solvent. From left to right initial as synthesized silica nanoparticles in lysine/water, ungrafted in methanol, ungrafted in acetonitrile, APDMES grafted in water at pH 2.

Initial efforts to amine functionalize the synthesized nanoparticles were problematic. Standard silane grafting methods^35^ on bulk silica, *e.g.* reaction of the silane in anhydrous toluene, led to irreversible aggregation. Upon removal of the organic solvent it was found that the particles could not be redispersed even with the aid of sonication. Prior literature^26^ suggests silica aggregation is essentially irreversible for sub-40 nm diameter particles even with grafting of functional groups on the surface, although it has been shown to be reversible for larger particles.^11^ This led to attempting to flocculate the particles then redisperse in an organic solvent without a drying step. Previously, it has been shown by De Roo *et al.*^38^ that amino acids can be utilized as versatile capping ligands for HfO_2_ and ZrO_2_ nanocrystals which at low pH allow for redispersion in methanol along with various other organic solvents. This method was then applied to the silica nanoparticle solution because native l-lysine was already acting as a capping group. The particles were flocculated using THF, centrifuged, then redispersed in methanol with the addition of TFA and sonication. Grafting of APDMES was then attempted in methanol, but the particle grafting resulted in only a small portion of particles being recoverable after centrifugation. However, the suspended particles could be further removed by adding toluene, but when it was attempted to redisperse in acidic water, the resulting suspension was cloudy and incomplete. This was likely due to incomplete grafting in methanol. Finally, grafting was then attempted in acetonitrile after first transferring ungrafted particles from methanol. Particles were flocculated from methanol using toluene then centrifuged and resuspended in acetonitrile with sonication. Grafting with APDMES in acetonitrile resulted in complete flocculation from solution and the particles could be recovered through centrifugation. The resulting particles could then be resuspended in water at a pH less than 5, with HCl used to reduce the pH. Scheme 1 shows a complete schematic of the synthesis, phase transfer, and amine grafting. Fig. 1 shows images of the suspended particles at each step of the process.

**Fig. 2:**
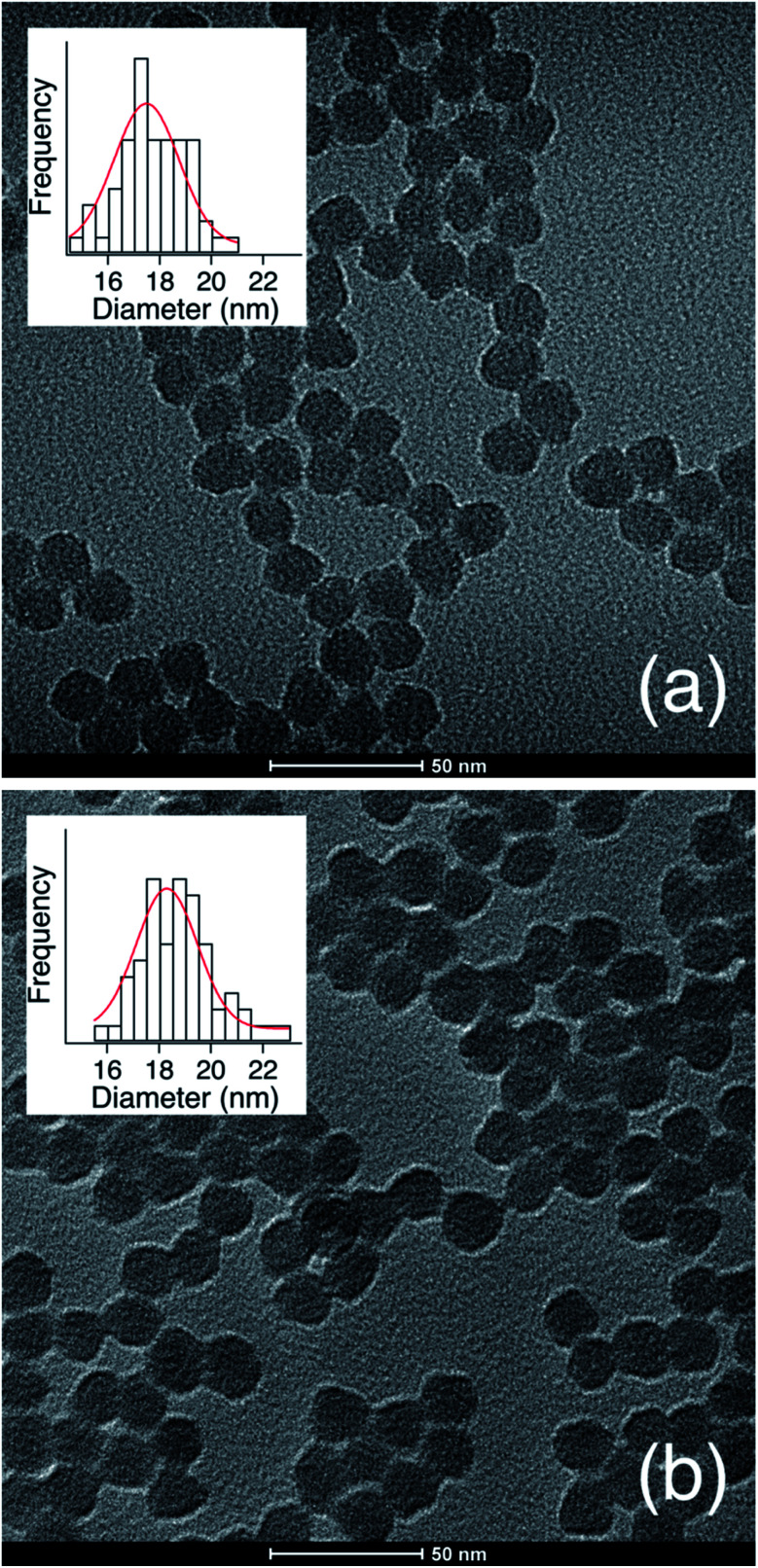
TEM micrograph and diameter distribution for (a) as synthesized silica nanoparticles and (b) APDMES grafted silica nanoparticles.

**Fig. 3:**
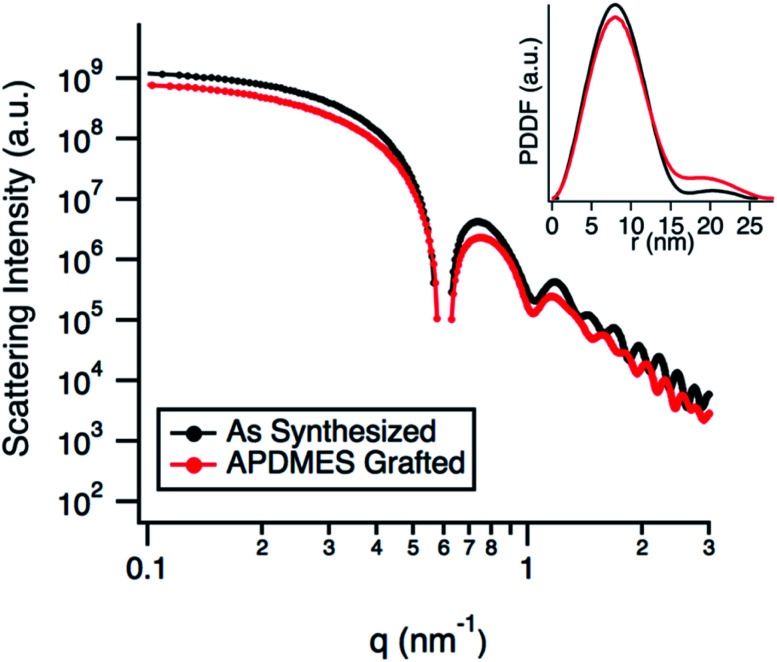
SAXS data comparing as synthesized silica nanoparticles to APDMES grafted particles. Inset shows the pair-distance distribution function (pddf).

TEM micrographs (Fig. 2b) show the g-NPs are between 15–20 nm in diameter with a mean diameter of 17.5 nm (±1.7 nm), which is very similar to the ungrafted nanoparticles with a mean diameter of 18.3 nm (±1.7 nm). However, TEM yields little information on the particle size when suspended in solution, which is the main focus of this study. SAXS was used to probe this and data obtained from a 1 wt% suspension of nanoparticles (Fig. 3) is consistent with TEM results. Both the full scattering curves and pddfs (pair distance distribution function) are essentially identical before and after grafting, with an average particle radius of 7.5 nm. The pddf gives a real space visualization of the scattering data and is the indirect Fourier transform of the scattering curve. The results demonstrate that the particles are completely redispersible as larger aggregates would contribute to the low-q region, *i.e.* there would be no plateau observed. DLS was also performed (Fig. S1†) for the grafted and pristine nanoparticles. There is a clear increase in diameter, about 17 nm for pristine compared to 22 nm for grafted, likely due to a number of factors. The pristine particles were at pH 9.7 due to the l-lysine and because the zwitterionic l-lysine was stabilizing the particles it could not be removed by dialysis or the pH reduced without particle agglomeration. In contrast, the grafted particles were at pH 2 and required acidic pH to be completely dispersed. These discrepancies in pH would lead to a different surface charge which could affect the hydrodynamic diameter. Further, the suspension viscosity was not measured directly but estimated to be that of pure water. Finally, because the APDMES ligands are covalently bound as opposed to l-lysine being capped on the surface, the APDMES ligand is immobilized and more rigid which could also lead to an increase in hydrodynamic diameter. The key point from the results above is that the TEM and SAXS are very self-consistent, while the DLS is qualitatively consistent likely due to some of the reasons described above.

A crucial aspect of the grafting process was to not allow particle agglomeration that would prevent redispersion by sonication. Irreversible agglomeration would occur when drying or mixing with a solvent that was not miscible with water, such as hexane or toluene. The most consistent results were obtained when performing the grafting in a solvent wherein particles were colloidally stable without the 3-aminopropyldimethyl-ethoxysilane (APDMES) but would flocculate from solution once grafted. However, it should be noted that the suspensions in methanol and acetonitrile resulted in significantly larger particle diameters in the range of 100–300 nm as seen in Fig. S2.† This shows that the particles can be grafted while slightly agglomerated and still be redispersed. It was also observed that particles could be flocculated by raising the pH to 10, then redispersed by reducing the pH back to 3. The DLS of before and after can be seen in Fig. S3.† It should also be noted that when grafting with (3-aminopropyl)trimethoxysilane (APTMS), as opposed to APDMES, the resulting particles diameter was around 35 nm, as seen in Fig. S4.† This is due to the formation of polymeric layers of silane from APTMS caused by residual water as the solvents and silanes were not rigorously dried and excess silane had to be used in all cases.

**Fig. 4:**
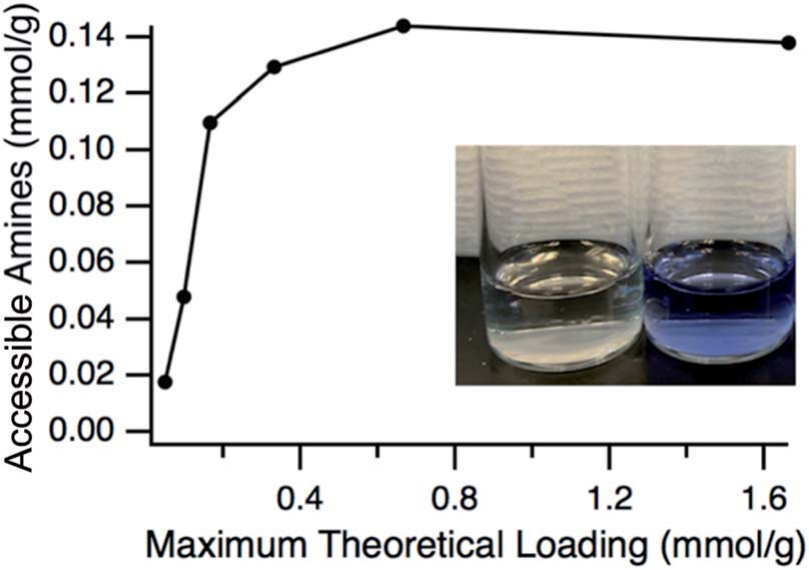
Quantification of accessible amine groups at different concentrations of APDMES added during synthesis. Ninhydrin was used as the amine reactive probe with solution colour shown after reaction for pristine and APDMES grafted shown in the inset.

Having verified that the functionalization process resulted in particles that have a very similar size to the pristine particles, the next step was to confirm the presence and accessibility of the grafted amines. This was confirmed by using two primary amine reactive probes, ninhydrin and fluorescamine. Ninhydrin is a pale-yellow colour but reacts with primary amines at elevated temperatures in alcohols to form a deep blue-purple complex in solution, known as Ruhemann's purple.^50,51^ This colour change is easily monitored with UV-vis spectroscopy with the absorbance maximum at approximately 580 nm. The accessible amine groups on the particles could then be calculated by comparison to a calibration curve. A comparison of ninhydrin solution exposed to ungrafted particles where the l-lysine had been exchanged for hexanoic acid and grafted particles can be seen in Fig. 4, with grafted showing the purple-blue complex and the ungrafted having no colour change. Hexanoic acid was used because any residual l-lysine would also react with ninhydrin. The amount of accessible amine groups at various amine loadings can be seen in Fig. 4, with a clear plateau reached at 0.14 mmol g^−1^ (∼0.1 amines per nm^2^). At high APDMES loadings the absence of the hexanoic exchange did not change the value, which shows that all l-lysine was replaced by APDMES. However, this maximum value is significantly lower than the theoretical loading based on surface area, which is cited as 2–3 aminosilane groups per nm^2^ for various types of silica.^52^ The discrepancy is likely due to the lower reaction yield for APDMES when compared to trimethoxysilanes, reduced accessibility due to slight aggregation of the particles in acetonitrile, and small amounts of water present during the grafting. However, the results show clear increases in amine content with increasing amine loading during the grafting step, confirming that APDMES can be grafted on the surface and the amount can be controlled. It should also be noted that the amine quantification using ninhydrin is not exact and previous literature^50^ has shown a 10–15% error associated with quantifying primary amine functional groups.

**Fig. 5:**
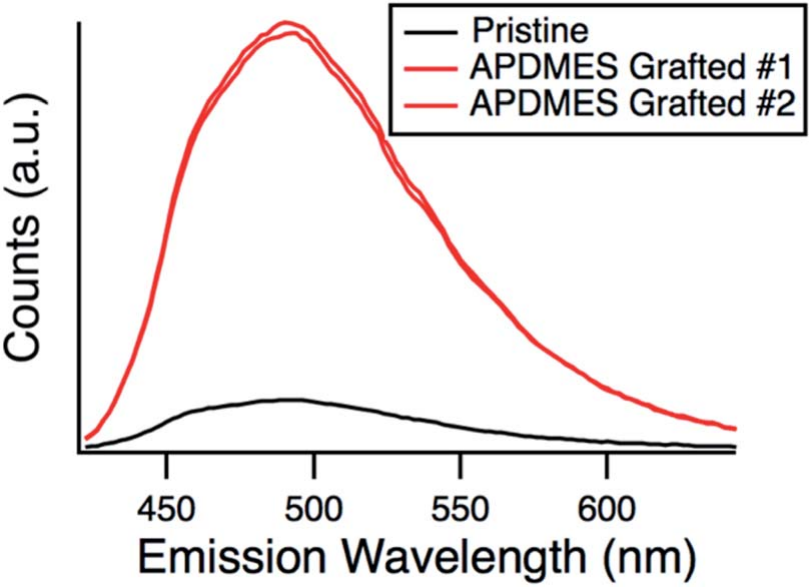
Fluorescence emission spectra for pristine and APDMES grafted silica particles after being exposed to fluorescamine.

Fluorescamine was used as a second method to confirm amine grafting on the surface while the particles were in solution. In contrast to ninhydrin which forms an amine complex in solution, fluorescamine reacts with a primary amine^53^ and the fluorescent complex is bound to the surface.^54^ The unreacted fluorescamine stays in solution and is not fluorescent. Fig. 5 shows the fluorescence wavelength scan of ungrafted hexanoic acid exchanged nanoparticles and APDMES grafted particles immediately after the addition of fluorescamine. Two separate experiments with the grafted particles were completed to show reproducibility. It can be seen that the fluorescent intensity is much higher for the grafted particles when compared to the ungrafted, which qualitatively confirms amine grafting. It should be noted that a potassium phosphate buffer was used to increase the pH to 8, suitable for the fluorescamine reaction to take place and because of this the particles flocculated from solution. Due to this the measurement was performed immediately after the buffer was added, although some particle aggregation and flocculation likely occurred over the time of the measurement.

**Fig. 6:**
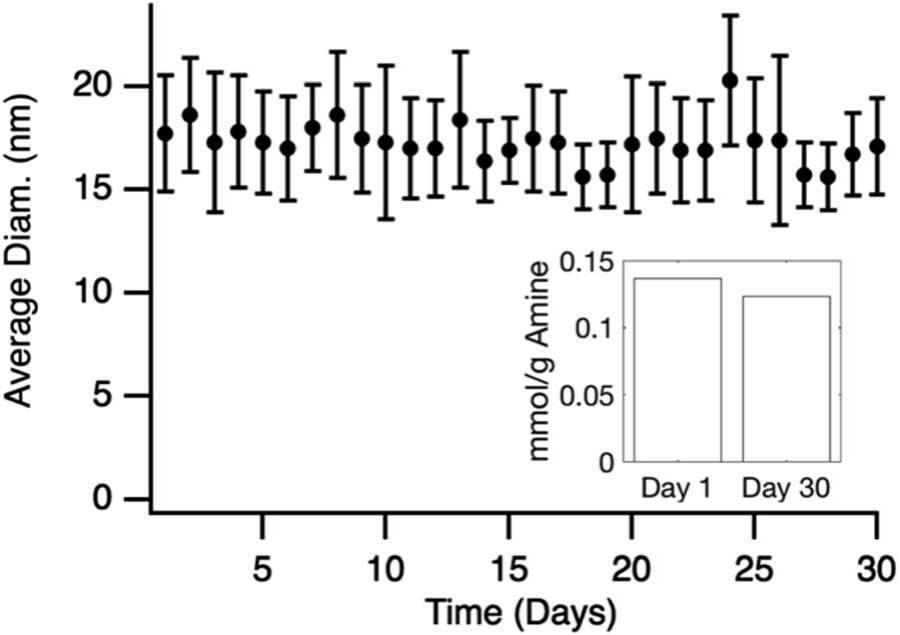
Average diameter of APDMES grafted silica nanoparticles calculated from DLS over 31 days. Inset shows ninhydrin quantification of amine groups after 30 days compared to day 1.

Grafted particles were tested for stability over time by measuring the effective hydrodynamic diameter (% by particle number) for an incubation period of 30 days as seen in Fig. 6. Results show the diameter stays constant over the time period with any deviation associated with the measurement due to DLS instrument error (Table S1†). Ninhydrin was then used to determine if there was any noticeable loss of amine groups on the surface. The calculated loading of 0.12 mmol g^−1^ was similar to the 0.14 mmol g^−1^ as calculated for the particles on day 1. TEM images (Fig. S5†) after the 30 day period also show no significant change in diameter. These combined results show that the particle size is stable in suspension and that there is not an appreciable loss of accessible amines over 30 days.

**Fig. 7:**
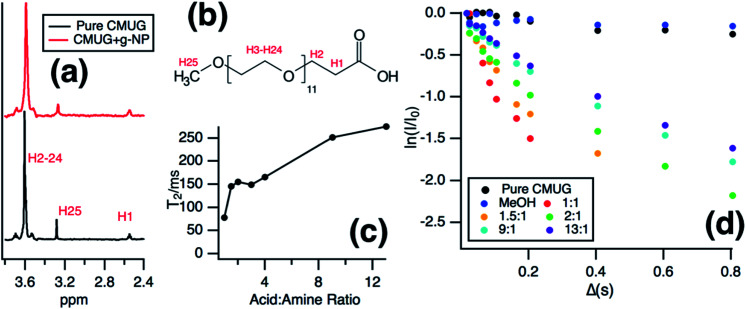
(a) NMR spectra of pure CMUG and CMUG in the presence of g-NPs at 1 : 1 CMUG : amine ratio. (b) CMUG molecule with labelled protons. (c) Estimated *T*_2_ values for increasing CMUG acid : amine ratios taken from the linear region below 150 ms. (d) *T*_2_ attenuation curves for increasing CMUG : amine ratios compared to pure CMUG and MeOH in the presence of g-NPs at 1 : 1 MeOH : amine ratio.

Solution NMR was then used to monitor the binding of the long chain water soluble organic acid, CMUG, to probe the g-NPs *in situ*. Organic acids are known to bind to amines^55–57^ when both are in the protonated states^58^ and the binding interaction was measured using a variety of solution NMR techniques to confirm amine loading and obtain mechanistic insights into amine-acid binding. The most direct way to measure the ratio of bound groups compared to free is to obtain a diffusion coefficient by using pulsed field gradient NMR,^59–61^ where the bound molecules exhibit the same diffusion coefficient as the particle. However, after calculating the diffusion coefficient from the PFG-NMR data, it was clear that only the free CMUG molecules could be observed (Fig. S6†). This was likely due to the very short spin–spin relaxation time, *T*_2_, for a 20 nm hydrodynamic diameter particle, which will edit out the bound signal^62,63^ during the PFG-NMR pulse sequence (*i.e. T*_2_ > *δ* + *τ*_r_ is required for the BPP STE sequence used). Previous literature has shown^62^ that nanoparticles with a hydrodynamic diameter above 15 nm have extremely short *T*_2_ relaxation times, below 2 ms, which would be even shorter than standard gradient pulse durations. It should also be noted that for the *T*_2_ NMR experiments only the terminal protons are shown, H25 in Fig. 7b, because the protons farthest from the nanoparticle surface have been shown^60,62^ to have the longest *T*_2_ due to increase mobility in solution. Fig. 7a shows the 1D NMR spectrum of neat CMUG as well as in the presence of amine grafted nanoparticle. The protons are labelled and can be seen on the CMUG molecule in Fig. 7b. Only one resonance could be observed for each proton, which is likely due to fast exchange or peak overlap, but line broadening could be observed for CMUG in the presence of the amine-grafted nanoparticles (Table 1). This gave rationale to study the differences of *T*_2_ relaxation in the presence of the particles and attempt to filter out the bound and free signals based on *T*_2_ differences. Additionally, Table S2† shows the comparison of the terminal protons to the other resonances with and without grafted nanoparticles. The resonances broaden significantly for the protons closer to the surface due to the very short *T*_2_. A sample decay curve (Fig. S7†) also shows a much faster relaxation rate for the non-terminal protons. *T*_2_ measurements were then performed which show that estimated relaxation of the terminal methyl protons (H25 in Fig. 7b) is much faster in the presence of the grafted particles (Fig. 7c). *T*_2_ significantly increases with increasing acid concentration (Fig. 7c), consistent with the CMUG:amine complex having a much smaller *T*_2_, *i.e.* faster relaxation. To explore this further a comparison of *T*_2_ values of CMUG terminal protons is shown alongside with methanol in Table 1 for stoichiometric amounts of probe molecule (*i.e.* 1 : 1 amine to probe molecule). In the case of methanol, a molecule which should not have as strong of an interaction with the amine groups the *T*_2_ value does not change significantly. By contrast a nearly 40-fold reduction in *T*_2_ is observed for CMUG.

**Table 1:** Calculated *T*_2_ and FWHM of probe molecules with and without g-NPs at 1 : 1 bound amine to probe molecule

Probe molecule		FWHM (Hz)	*T*_2_ (s)
CMUG	Pure	2.78	3.31
	g-NP	7.79	0.08
MeOH	Pure	1.95	7.08
	g-NP	2.08	5.05

The signal decay from echo delay of the *T*_2_ measurement's (Fig. 7c) also shows a clear nonlinearity which is evidence of a two-state system of bound and unbound CMUG. This nonlinearity is common in systems where only one resonance is observed for both bound and unbound states.^59,64^ As the concentration increases, the fit becomes closer to linear as the signal is shifted to almost completely unbound molecules, although since there is still exchange the *T*_2_ value at higher concentration is still lower than pure CMUG. Likely this is due to secondary interaction outside of the main binding of the acid and amine at the surface. Due to this the *T*_2_ value for the CMUG with g-NPs was calculated from echo delays below 150 ms to include as much contribution of the bound state as possible. A comparison of linear fits below 150 ms and of every delay time point can be seen in Fig. S8 and Table S3.† However, it should be noted that the bound signal could never be completely isolated because of the presence of free molecules in a fast exchange system. Additionally, the estimated *T*_2_ values of the bound linear regime are likely much larger than the true bound species, as it has been reported that a particle of similar diameter would likely have a much smaller *T*_2_.^62^ Further experiments are being conducted to more accurately distinguish between the bound and unbound states. However, the current results show a clear difference in *T*_2_ when grafted particles are present indicating the amine groups are accessible when suspended.

## Conclusions

Colloidal silica nanoparticles were grafted with amines by using a phase transfer method that allows for silica nanoparticle dispersion in acetonitrile. Once suspended in acetonitrile, the silica nanoparticles could be grafted with APDMES and easily redispersed in acidic water by sonication. The resulting particles have approximately the same diameter as the ungrafted parent silica particles as confirmed by SAXS and TEM. Primary amine groups were confirmed on the surface by both ninhydrin and fluorescamine analysis, where clear differences were observed when compared to the ungrafted nanoparticles. The grafted nanoparticle suspension was also shown to retain colloidal stability over an incubation period of a month with no diameter change observed by DLS and minimal amine loss when the amine groups were quantified on the surface using ninhydrin.

*In situ* solution NMR of the amine grafted nanoparticles provides additional proof the accessibility of the amine groups due to the *T*_2_ attenuation of CMUG in the presence of the amine grafted particles. Analysis of the signal attenuation also showed a curve with two distinct slopes, suggesting observation of a bound and free CMUG state. This allowed for calculation of *T*_2_ values and gave mechanistic insight into organic acid–amine binding process at the molecular level.

## Conflicts of interest

The authors declare no conflicts of interest.

## Supplementary Material

NA-002-C9NA00772E-s001
