# Supplementary material for: Covalently functionalized uniform amino-silica nanoparticles. Synthesis and validation of amine group accessibility and stability
Source: Nanoscale Adv. 2020 Jan 16;2(2):860–8. doi: 10.1039/c9na00772e (PMC9417799; doi:10.1039/c9na00772e)
Supplement: NA-002-C9NA00772E-s001 [file NA-002-C9NA00772E-s001.pdf]

Electronic Supplementary Material (ESI) for Nanoscale Advances.
This journal is © The Royal Society of Chemistry 2020

Supporting Info

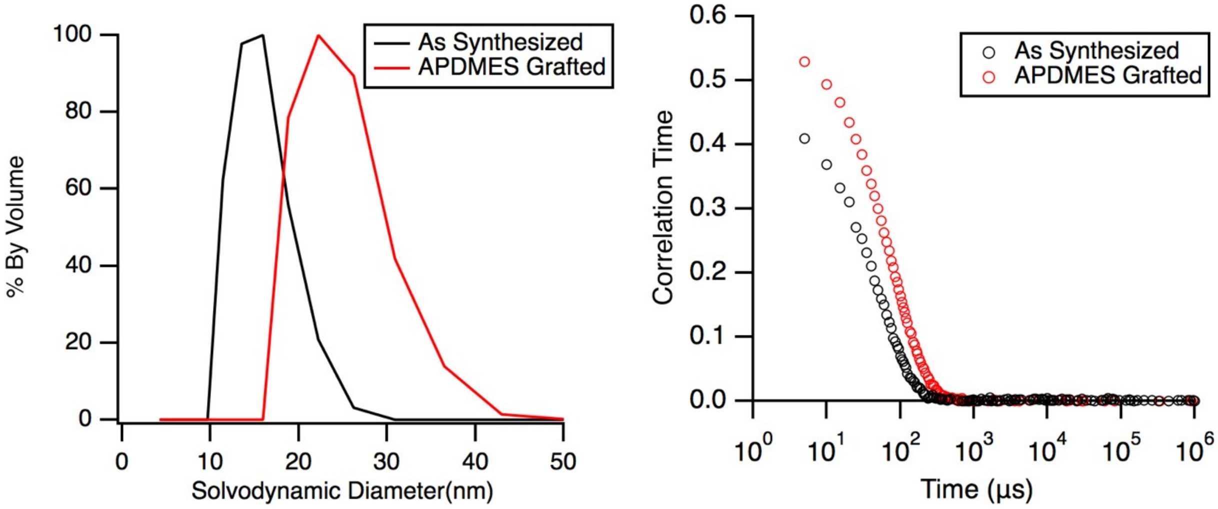

Figure S1. (a) DLS diameter and (b) corresponding correlogram comparing as synthesized pristine nanoparticles and APDMES grafted in water at 1 wt%.

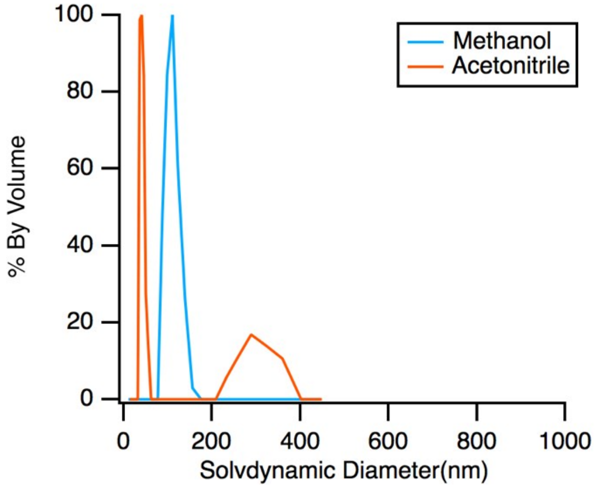

Figure S2. DLS results of particles transferred into methanol and acetonitrile. The measurements were completed directly after sonication.

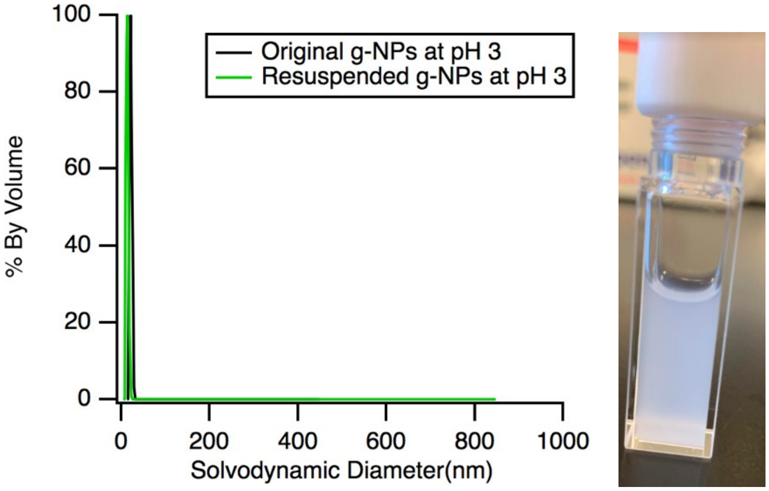

Figure S3. (Left) DLS of amine grafted nanoparticles at pH 3 compared to the particles after the pH was raised to 11 when flocculation occurred, then reduced back to pH 3 and resuspended with sonication. Axis shown to 1000 nm to show no aggregation. **(Right)** Image of particles flocculated at pH 11.

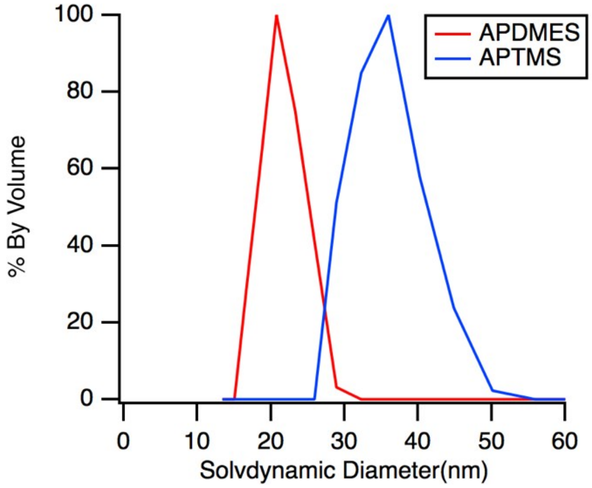

Figure S4. Comparison of diameter of particles grafted with the same molar amount of (3-aminopropyl)trimethoxysilane (APTMS) and (3-aminopropyl)dimethylethoxysilane (APDMES) in water, both at 1 wt%.

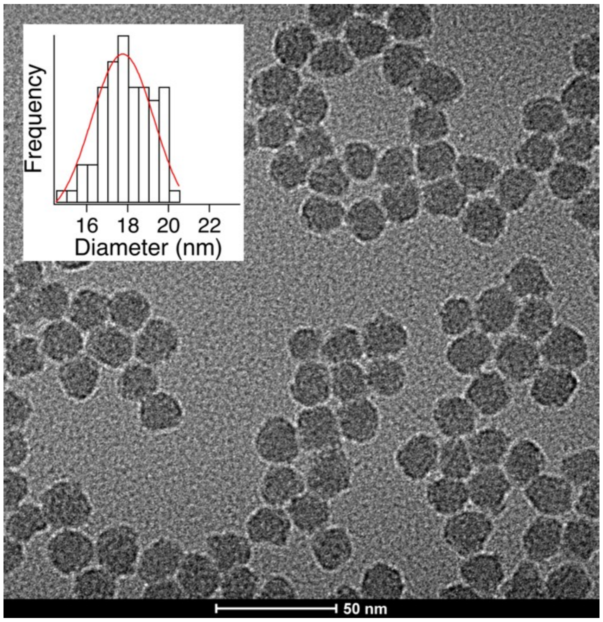

Figure S5. TEM micrograph with corresponding diameter distribution of g-NPs after a 30-day incubation period.

Table S1. Error of 5 runs of the same g-NP sample for DLS calculations.

Sample	Effective Diameter (nm)	Polydispersity
1	19.4	0.291
2	15.6	0.321
3	17	0.274
4	16.2	0.289
5	18.7	0.242
Avg.	17.4	0.283
Std Dev.	1.5	0.025

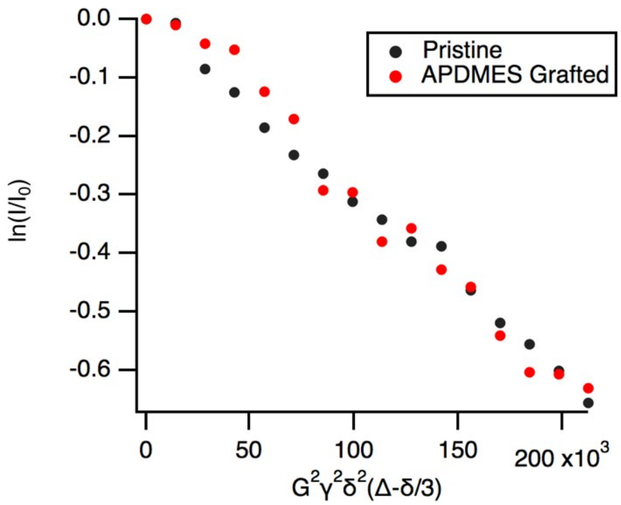

Figure S6. PFG-NMR gradient attenuation for pristine CMUG compared to in the presence of g-NP. The calculated diffusion coefficient for both were around 320 µm/s^2 with no significant differences. Acid:amine ratio is 1:1.

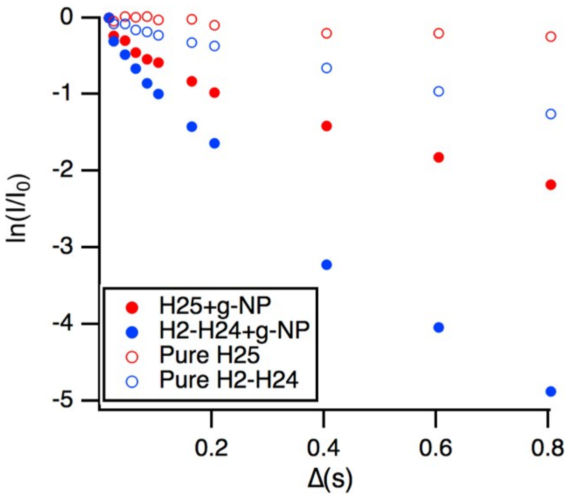

Figure S7. T_2 decay comparing the terminal protons (H25) to the middle protons(H2-H24) of CMUG with and without grafted nanoparticles. Acid:amine ratio is 2:1. It can be observed that the middle protons have a much faster T_2 and the initial linear region is very short as a result.

Table S2. Peak widths for all protons on CMUG with and without grafted nanoparticles.

		H1	H2-H24	H25
FWHM (Hz)	Pure	4.14	4.96	2.78
	g-NP	15.62	12.16	7.79

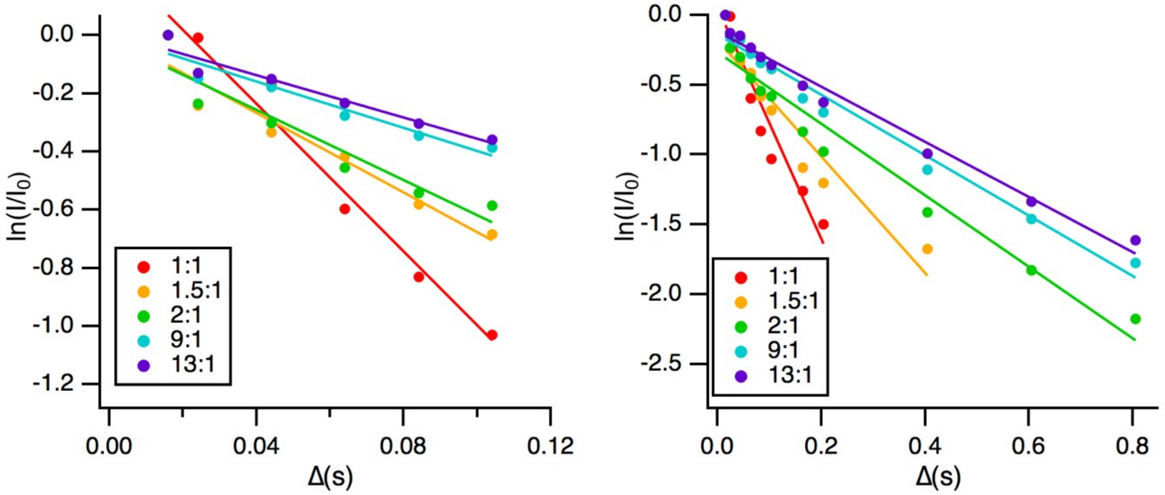

Figure S8. (a) Linear fit to T_2 attenuation data under a delay time of 150 ms and (b) linear fit to T_2 attenuation of all points.

Table S3. Calculated T_2values from linear fits under 150 ms delay time compared to full fits.

Acid to Amine	Calculated T_2/ms Under 150 ms	Calculated T_2/ms Full Fit
1 to 1	78	120
1.5 to 1	146	239
2 to 1	155	390
9 to 1	252	461
13 to 1	275	505
